# Subtle Phenotype Differences in Psychiatric Patients With and Without Serum Immunoglobulin G Antibodies to Synapsin

**DOI:** 10.3389/fpsyt.2019.00401

**Published:** 2019-06-07

**Authors:** Sverre Georg Sæther, Arne Vaaler, Anita Evjenth, Therese Aune, Markus Höltje, Klemens Ruprecht, Morten Schou

**Affiliations:** ^1^Division of Mental Health, St. Olav’s Hospital, Trondheim University Hospital, Trondheim, Norway; ^2^Department of Mental Health, Norwegian University of Science and Technology, Trondheim, Norway; ^3^Institute of Integrative Neuroanatomy, Charité—Universitätsmedizin Berlin, corporate member of Freie Universität Berlin, Humboldt-Universität zu Berlin, and Berlin Institute of Health, Berlin, Germany; ^4^Department of Neurology, Charité—Universitätsmedizin Berlin, corporate member of Freie Universität Berlin, Humboldt-Universität zu Berlin, and Berlin Institute of Health, Berlin, Germany

**Keywords:** synapsin, antibodies, psychiatric disorders, autoimmunity, serum

## Abstract

The discovery that antibodies targeting neuronal antigens can induce severe psychiatric symptoms has been a significant progress in the understanding of psychiatric disorders. Antibodies targeting synapsin I in serum and cerebrospinal fluid (CSF) were first reported in 2015 in a patient with limbic encephalitis. Because of its regulatory function for neurotransmitter release, synapsin I has been suggested to play a role in psychiatric disorders. It is, however, unknown whether or not synapsin antibodies are of clinical significance in patients with psychiatric disorders. In the present study, we aimed to investigate if synapsin I immunoglobulin (Ig)G serum antibody positive patients admitted to acute psychiatric care have a different psychiatric phenotype than synapsin IgG antibody negative patients. A total of 13 anti-synapsin positive patients were matched for age, sex, and psychiatric diagnosis with 39 anti-synapsin negative patients from the same cohort. The groups were compared regarding 11 clinical features frequently seen in anti-neuronal antibody associated disorders. Anti-synapsin positive patients had higher agitation scores as measured with the Positive and Negative Syndrome Scale Excited Component [median (interquartile range) 11 (8) versus 7 (7), *p* = 0.04] compared to controls. However, the absolute scores were low in both groups, and the difference may not be clinically significant. Other clinical features assessed (e.g. hallucinations, delusions) did not differ between groups. We conclude that synapsin serum IgG antibodies lack syndrome specificity in patients admitted to acute psychiatric inpatient care. However, further studies addressing functional effects of synapsin antibodies are needed to conclude whether or not they have a pathophysiological relevance.

## Introduction

A large amount of research has been carried out to unravel the possible significance of anti-neuronal antibodies for patients with severe psychiatric disorders (1). Antibodies targeting synaptic and neuronal membrane antigens [e.g. anti-*N*-methyl *d*-aspartate receptor (NMDAR)] have gained most attention, mainly because of their close association to encephalitic syndromes with prominent psychiatric symptomatology and good response to immunotherapy (e.g. NMDAR encephalitis) (2).

Antibodies directed at hitherto unknown neuronal antigens continue to be discovered. In 2015, a patient with limbic encephalitis with immunoglobulin (Ig)G and IgA antibodies targeting synapsin I in serum and cerebrospinal fluid (CSF) was reported (3). Following up on this finding, we recently found synapsin I IgG antibodies in serum in subgroups of patients with different psychiatric and neurological disorders; including 13 out of 207 patients (6.3%) admitted to acute psychiatric inpatient care (4).

Synapsin I is a pre-synaptic phosphoprotein that belongs to one of the largest families of synaptic proteins (the synapsins). Both isoforms (synapsin Ia and Ib) play important roles in the formation of synapses and modulation of neurotransmitter release (5), suggesting potential roles in psychiatric disorders. Indeed, authors of previous studies have reported that patients with schizophrenia (6) and bipolar disorder (7) have lower levels of synapsin I in the hippocampus compared to controls. It remains, however, unknown if antibodies targeting synapsin are of clinical significance in psychiatric patients.

The characterization of clinical phenotypes (syndromes) associated with specific anti-neuronal antibodies (e.g. anti-synapsin) is important for several reasons. For instance, syndrome specificity would increase the likelihood that the antibody is clinically relevant (i.e. important for diagnostic or treatment-related considerations). The presence of anti-synapsin IgG antibodies in patients with different psychiatric disorders (4) argues against any diagnostic specificity. However, since symptoms overlap between psychiatric disorders it is possible that anti-synapsin positive patients have a phenotype that is not captured by traditional psychiatric diagnostic entities.

In the present study, we therefore aimed to compare the psychiatric phenotype in patients with and without synapsin IgG antibodies admitted to acute psychiatric care. We hypothesized that anti-synapsin positive patients have a higher frequency and severity of psychiatric symptoms seen in autoimmune encephalitic syndromes as compared to anti-synapsin negative patients.

## Materials and Methods

### Setting

This is a single-center case–control study (St. Olavs Hospital, Trondheim University Hospital, Trondheim, Norway). The facility receives all adult patients (>18 years) in need of acute psychiatric inpatient care in the catchment area (the city of Trondheim and surrounding area).

### Patients

During September 2011–March 2012, 340 out of 654 admitted patients (52%) consented to donate blood for future research. The only inclusion criterion was admission to acute psychiatric inpatient care. Patients were excluded if they were discharged before consent could be obtained or lacked ability to consent or proficiency in Norwegian or English. Out of the 340 participants, 207 patients were selected on the basis of an International Classification of Diseases (ICD)–10 diagnosis (8) of schizophrenia spectrum disorder (F20–F29, *n* = 46), depressive disorders (F32–F33, *n* = 81) or bipolar disorder (F31, *n* = 59), or because they previously had tested serum positive to other anti-neuronal antibodies (*n* = 41) (9).

This subgroup was tested for the presence of serum IgG antibodies targeting synapsin Ia and Ib, as previously reported (4). A total of 13 out of 207 patients (6.3%) were positive to synapsin Ia and/or Ib antibodies in serum (4).

In the present study, the 13 anti-synapsin positive patients were matched (1:3) for sex, age, and psychiatric diagnosis with 39 anti-synapsin negative controls from the same cohort.

### Variables

The two groups were compared with respect to baseline demographics (**Table 1**) and clinical characteristics (**Table 2**).

**Table 1:** Demographic and clinical data of anti-synapsin positive and negative patients.

Demographic and clinical characteristics	Anti-synapsin + (*n* = 13)	Anti-synapsin − (*n* = 39)	*p* value^b^
Sex (n (%) males)	6 (46.2)	18 (46.2)	1.00^c^
Age (mean [SD])	44.6 (18.6)	45.4 (17.0)	0.86^d^
Main ICD-10 diagnosis, n Alcohol or substance use (F10–19) Schizophrenia spectrum (F20–29) Bipolar disorders (F31) Depressive disorders (F32–33) Personality disorders (F60–61)	2 2 2 6 1	3 6 8 19 3	0.95
Psychopharmacological treatment, *n* (%)^a^ Antipsychotics Antidepressant Mood stabilizers Benzodiazepines	2 (15.4) 3 (23.1) 1 (7.7) 3 (23.1)	10 (26.3) 11 (28.9) 5 (13.2) 6 (15.8)	0.71 1.00 1.00 0.68

ICD-10, International Classification of Diseases-10; SD, standard deviation.

^a^Missing data on 1 control. ^b^Fisher’s exact test if not otherwise specified. ^c^chi square test. ^d^Mann–Whitney U test.

**Table 2:** Comparison of clinical characteristics in anti-synapsin positive and negative patients.

Clinical characteristics	Anti-synapsin + (*n* = 13)	Anti-synapsin − (*n* = 39)	*p* value^f^
Acute/sub-acute onset, *n* (%)	5 (38.5)	16 (41.0)	1.00^g^
Hallucinations, *n* (%)	4 (30.8)	10 (25.6)	0.73
Delusions, *n* (%)	4 (30.8)	11 (28.2)	1.00
Irritability, *n* (%)^a^	3 (23.1)	7 (19.4)	1.00
Disorientation, *n* (%)^b^	2 (15.4)	2 (5.7)	0.29
Disinhibition (median, IQR)	2 (1, 4)	1 (1, 3)	0.15^h^
Agitation (median, IQR)	11 (7.5, 15.5)	7 (6, 13)	0.04^h^
Symptom fluctuation (median, IQR)^c^	3.0 (1, 5)	1.0 (1, 3)	0.09^h^
Motor retardation (median, IQR)^d^	10.0 (8, 10)	10.0 (8, 10)	1.00^h^
Increased motor activity (median, IQR)^c^	1.0 (1, 3)	1.0 (1, 3)	0.72^h^
History of motor seizures, *n* (%)^e^	4 (40)	7 (20)	0.23

IQR, Interquartile range.

Missing data on: ^a^three controls. ^b^four controls. ^c^two cases and three controls. ^d^two cases and four controls, ^e^three cases and four controls. ^f^Fisher’s exact test if not otherwise specified. ^g^chi square test. ^h^Mann–Whitney U test.

Information on baseline demographics (age, sex, psychiatric diagnosis, and use of psychopharmacological medication) was collected during index admission. Psychiatric diagnosis was set in a consensus meeting according to the ICD-10 criteria for research (8). At least two psychiatrists and/or senior clinical psychologists participated in the evaluation together with the physician or psychologist in charge of the treatment. The physician on call recorded the use of psychopharmacological medication at admission (before serum sampling); this information was later converted to dichotomous variables (e.g. neuroleptics yes/no).

The 11 variables (clinical characteristics) included in the present study were selected on basis of their relevance for other anti-neuronal antibody associated disorders [systematic literature search, *see* Ref. (10)], and their availability in the dataset (prospectively obtained) or through a chart review (retrospectively obtained). The investigators performing the chart review (TA and AE) were blinded for antibody status.

Eight variables were obtained prospectively during the index admission. The Brøset Violence Checklist (BVC) was used to assess the presence of irritability and disorientation (yes/no) (11). The positive and negative syndrome scale—excited component (PANSS-EC, score 5–35) and PANSS Item G14 (score 1–7) was used to assess agitation and disinhibition (12). The degree of symptom fluctuation, motor retardation, and increased motor activity were evaluated using SOMAS Item A, B, and C (score 1–10), respectively (13). For PANSS-EC, PANSS Item G14 and Symptomatic Organic Mental Disorder Rating Scale Item A and C a higher score indicates more severe symptomatology, whereas for SOMAS Item B the scale is inverted (a lower score indicate more severe symptomatology). A self-report questionnaire filled out during admission assessed whether or not the patient had a history of motor seizures (yes/no).

Three variables were obtained by a retrospective blinded chart review [acute/subacute onset (<3 months, yes/no), presence of hallucinations (all modalities, yes/no), and delusions (yes/no)].

### Antibody Analyses

Sera (dilution 1:320) were screened for anti-synapsin IgG using HEK293 cells transfected with human synapsin Ia and Ib. Patients whose sera contained IgG antibodies to human synapsin Ia and/or Ib at a titer of 1:320 were considered to be synapsin antibody positive and were included as cases in the present study (4).

### Statistics

We performed group comparisons of anti-synapsin positive and negative patients on the selected variables. Categorical variables were analyzed using Fisher’s exact test (if > 0 cells had an expected count < 5) or chi square test. Continuous variables were compared using the Student’s *t*-test (if data were normally distributed) or Mann–Whitney U-test (if data were not normally distributed). The distribution (normality yes/no) was evaluated by Shapiro–Wilk test and inspection of histogram. The significance level was set at 0.05. No formal adjustment for multiple testing was made. Effect sizes were calculated for statistically significant findings; Mann–Whitney U test *r* = *z*/√*N* (a value of 0.1 is considered a small effect, 0.3 a medium effect, and 0.5 a large effect) (14). SPSS version 25 for Mac was used for statistical analysis.

## Results

Age, sex, and psychiatric diagnosis and use of psychopharmacological medication at admission did not differ significantly between cases and controls (**Table 1**).

Anti-synapsin positive patients had a statistically significant higher degree of agitation [median (interquartile range) 11 (8) versus 7 (7), *p* = 0.04] compared to anti-synapsin negative controls (**Table 2**). This corresponded to an effect size of *r* = 0.28. None of the other variables differed statistical significantly between the groups.

## Discussion

The main finding in this study is that the psychiatric phenotype is rather similar in serum anti-synapsin IgG positive and negative patients admitted to acute psychiatric inpatient care. Specifically, anti-synapsin positive patients did not have a higher frequency of acute/subacute symptom onset, hallucinations, delusions, irritability, disorientation, or history of seizures as compared to anti-synapsin negative patients. The two groups also had comparable degrees of disinhibition and motor activity.

The only statistically significant difference between the two groups was a higher degree of agitation in anti-synapsin positive patients {PANSS-EC scores, median [interquartile range (IQR)] 11 [7.5, 15.5] versus 7 [6, 13], *p* = 0.04}. PANSS-EC is validated for measuring agitation in acute settings, but has often been used in more homogenous patients samples with higher levels of agitation compared to our cohort (12, 15). Agitation is often seen in patients with autoimmune encephalopathies (16). It is therefore interesting to note the statistically significant difference in agitation between the case and control group. However, the effect size in moderate (*r* = 0.28), and the absolute difference is small and not possible to use to identify individual cases. We therefore question the clinical significance of this finding, but recommend assessing agitation in future studies exploring the significance of anti-neuronal antibodies in psychiatric patients.

A statistical trend towards more symptom fluctuation (as measured by SOMAS Item A) in the case group was also observed. The median score in the case group [3 (IQR 1, 5)] corresponds to “minor changes in symptoms during the past 24 hours” whereas the score in the control group [median 1 (IQR 1, 3)] corresponds to “complete symptom stability during the past 24 hours.” Symptom fluctuation is an important feature in autoimmune encephalopathies (17) and other organic psychiatric syndromes (e.g. delirium), and should be assessed in future studies investigating the psychiatric phenotype of organic brain disorders. However, the finding in this study allows no conclusion of its relevance for the psychiatric phenotypes seen in anti-synapsin positive patients.

The study has some limitations. First, the lack of correction for multiple testing could have resulted in type I statistical errors; that is to claim differences that are not there. However, we argue that the only statistical significant finding (increased agitation score in cases) may not be clinical significant. Second, the low number of cases may leave the study underpowered to detect minor differences between the groups. Third, the use of self-report questionnaire (one variable) and retrospective chart review (three variables) could make the study prone to recall and report bias. Fourth, the use of dichotomous variables (yes/no) may be insufficient to classify complex phenomena such as hallucinations and delusions. Lastly, it is important to note that we only included patients with serum synapsin IgG antibodies in this study. We cannot rule out that serum IgA antibodies or CSF IgA and/or IgG synapsin antibodies have a higher clinical relevance (the index patient reported in 2015 had both IgA and IgG synapsin antibodies in serum and CSF) (3).

In conclusion, the observed similarities in psychiatric diagnoses (4) and symptomatology (this study’s main finding) in anti-synapsin serum IgG positive and negative patients suggest that synapsin serum IgG antibodies lack syndrome specificity in patients admitted to acute psychiatric care. However, phenotypic similarities are not sufficient evidence to conclude that synapsin antibodies lack clinical significance. Future studies should address functional effects of antibodies to synapsin to better clarify their potential pathophysiological relevance.

## Ethics Statement

The study was conducted in accordance with the Declaration of Helsinki and approved by the regional committee for medical and health research ethics, central Norway (2011/137). All participants provided written informed consent.

## Author Contributions

SS and MS designed the study and performed the statistical analyses. SS drafted the manuscript. AV, MS, AE, and TA collected clinical data. KR and MH performed the laboratory analyses. All authors were involved in revising the manuscript for intellectual content. All authors read and approved the final manuscript.

## Funding

This study was supported by the Research Fund of Charité–Universitätsmedizin Berlin, Berlin, Germany and the Central Norway Regional Health Authority.

## Conflict of Interest Statement

The authors declare that the research was conducted in the absence of any commercial or financial relationships that could be construed as a potential conflict of interest.
