# Supplementary material for: Subtle Phenotype Differences in Psychiatric Patients With and Without Serum Immunoglobulin G Antibodies to Synapsin
Source: Front Psychiatry. 2019 Jun 7;10:401. doi: 10.3389/fpsyt.2019.00401 (PMC6567930; doi:10.3389/fpsyt.2019.00401)
Supplement: Supplementary file 1 [file Image_1.pdf]

The Symptomatic Organic Mental Disorder Rating Scale (SOMAS).

The ratings of all items are based on the investigator's personal examination of the patient, and/or information in the case records and/or information from the hospital ward staff.

The scorings are 1 - 10 on the following 5 items:

A: Degree of observable change in symptoms during the previous 24 hours

1: The symptoms have been completely stable throughout 24 hours.

3: Minor changes in symptoms during the past 24 hours (e.g., increased symptoms in the morning as in a depressive episode).

5: Some change of symptoms (e.g., breakthrough of depressive symptoms in hypomania).

8: Frequent alternation of symptoms, dominating more than half of the day.

10: Rapid fluctuation of symptoms from one half hour to the next.

B: Degree of motor retardation, rated during the period or periods of the previous 24 hours in which the patient was most depressed.*

1: The patient has been almost completely immobile and virtually unresponsive to external stimuli.

3: Movements are extremely slow, resulting in a minimum of activity and speech. The patient is mostly sitting idly or lying down.

5: The patient has slow movements, and speech may be characterized by poor productivity, including long response latency, extended pauses, or slow pace.

8:   Slight diminution in rate of movements and speech.

10: No motor retardation.

C: Degree of increased motor activity, rated during the period or periods the previous 24 hours when the patient was most depressed.*

1: No increased motor activity.

3: The patient is slightly agitated with hypervigilance or has a tendency towards mild overarousal. The speech is slightly pressured.

5: The patient is clearly agitated and overaroused with affected speech and motor activity.

8:   Marked excitement dominates the period and restricts attention and vital functions such as eating and sleeping.

10: The excitement is so extreme that interpersonal interaction is virtually impossible. The patient has acceleration of speech and motor activity resulting in incoherence and exhaustion.

D: Degree of patient's insight into his or her condition/symptoms

1: Mature and thoroughly considered attempt at explaining the condition. This explanation may or may not be psychotic.

3: The patient has been thinking of various possible explanations and has come up with a well-founded opinion about some of them.

5: The patient wonders about different causes of the condition, but is unsure.

8: The patient has one or several ideas about the cause, without any considered argumentation.

10: Patient is totally bewildered to what has happened or to what causes the condition.

X: Not possible to score; e.g. due to incapability to communicate verbally.

E: Degree of the patient's concern in finding an explanation for his or her condition/symptoms

1: Considerable engagement in finding an explanation of the condition.

3: Moderate engagement in finding an explanation of the condition.

5: Some engagement in finding an explanation of the condition.

8: Minimal engagement in finding an explanation of the condition.

10: The patient does not wonder at all what may have caused the condition.

X: Not possible to score; e.g. due to incapability to communicate verbally.

* Items B and C are modifications of two PANSS items (Kay SR, Fiszbein A, Opler LA. The Positive and Negative Syndrome Scale (PANSS) for schizophrenia. Schiz Bul 1987;13:261-76.)

Ref: *Vaaler AE, Morken G, Iversen VC, Kondziella D, Linaker OM. BMC Neurol. 2010 Jul 30;10:67.PMID: 20673344*
